# Musculoskeletal diagnostic ultrasound imaging for thickness measurement of four principal muscles of the cervical spine -a reliability and agreement study

**DOI:** 10.1186/s12998-016-0132-9

**Published:** 2017-01-04

**Authors:** Cecilie Krage Øverås, Birgitte Lawaetz Myhrvold, Gro Røsok, Eli Magnesen

**Affiliations:** 1 MSc Chiropractic, MSc Ultrasound (Musculoskeletal), NEMUS Trondheim, Fjordgata 80, 7010 Trondheim, Norway; 2 MSc Health Sciences, Clinical Biomechanics, MSc Ultrasound (Musculoskeletal), NEMUS Ullevål, Sognsveien 75B, 0855 Oslo, Norway; 3 DC, MSc Ultrasound (Musculoskeletal), Kiropraktorhuset Elverum, Storgata 7b, 2408 Elverum, Norway; 4 DC, MSc Ultrasound (Musculoskeletal), Kiropraktorklinikken Holmestrand, Havnegaten 23, 3080 Holmestrand, Norway

**Keywords:** Diagnostic ultrasound, Reliability, Cervical spine/neck muscles, Longus colli, Deep cervical extensors, Semispinalis capitis, Rectus capitis posterior major, Intra-class correlation coefficient, Bland-Altman’s limits of agreement, Linear regression analysis

## Abstract

**Background:**

The reliability of musculoskeletal diagnostic ultrasound imaging (MSK-DUSI) for the evaluation of neck musculature has been sparsely documented in the research literature. Until now, research has featured a limited number of subjects and only few studies have tested for both inter- and intra-reliability using appropriate methodology.

**Methods:**

Four examiners conducted an inter- and intra-rater reliability and agreement study. Fifty females with and without neck pain (NP) between the ages of 20–70 were recruited from October 2014 to April 2015. The muscles that were evaluated were the longus colli (Lcol), the rectus capitis posterior major (Rcpm), the deep cervical extensors (Dce) and the semispinalis capitis (Sscap). Each of the examiners captured ultrasound images of their allocated muscle and measured the thickness of that muscle twice, on separate occasions, for the first part of the intra-rater reliability study. For the second part, a second image of the same muscle was taken on the same subject and measured by the same examiner. The four examiners then met to measure on each other’s images, to test inter-rater reliability. Their results were compared pair-wise using Interclass Correlation Coefficients (ICC) and Bland-Altman plots. Linear regression analysis was performed to evaluate for possible bias.

**Results:**

Inter-rater reliability was found to be good for Lcol and Sscap muscles and moderate towards poor for the deeper Rcpm and Dce muscles. Intra-rater reliability was good for all the muscles, with the exception of the Dce, which was found to be moderate in the second part of the study. The B&A plots showed good agreement, few outliers, and no bias. However, the agreement intervals indicated a measurement error within the variance of the method that may not have been acceptable for these small muscles if the aim is to evaluate change in thickness.

**Conclusions:**

This study found that MSK-DUSI had variable reliability in assessing the thickness of the Lcol, Rcpm, Dce, and Sscap muscles. No bias was demonstrated, but agreement intervals were wide.

## Background

### Neck pain and muscle function

Neck pain (NP) is common and represents a disabling and costly problem to society [1]. It is a global health problem ranked as the fourth most common cause of loss of quality of life and incapacity [2]. The recovery rate tends to be low and the recurrence of neck pain episodes is common, demonstrating the persistence of neck pain [3–8]. Altered muscle function and morphological changes in these muscles are recognised features of painful neck disorders [9–14]. The underlying mechanisms of these changes and the implications for functional and physical impairment are not fully understood. Neck pain may occur in an impaired cervical motor system, where functional demands exceed physiological capacity [13]. This is thought to be a factor in the persistent or recurrent nature of mechanical NP [15] and suggests that clinicians ought to incorporate the evaluation of muscle function into their diagnostic considerations. Still, there is a need to establish valid, reliable and useful clinical and biological markers of neck dysfunction [16].

The longus colli (Lcol) function as a stabiliser and flattener of cervical lordosis [10, 17]. The sub-occipitals contribute to finer movement and stabilisation [18], particularly the rectus capitis posterior major (Rcpm) since it crosses the two upper cervical joints [10]. The deep cervical extensors (Dce), gives proprioceptive feedback and are considered important for intervertebral segmental control [10, 19–23]. The intramuscular function of the Semispinalis capitis (Sscap) is not completely clear, but it exerts large extensor movement to the head and neck [15]. Altered muscle activation, atrophy, increased fatty infiltration and decreased muscle strength has been reported in these muscles [24–27] and they seem to play a role in cervicocephalic – and tension type headaches, whiplash-mechanism-related complaints, post fusion surgery, work-related and long-lasting NP [25, 26, 28–32].

In recent years, MSK-DUSI has emerged as a method to evaluate morphological changes within neck muscles [33]. The amount of change in thickness at rest and during isometric contraction is considered an indirect measure of muscle function [34]. MSK- DUSI may hence play a role in the subgrouping process for diagnosis of NP, but also to evaluate effects of interventions. For diagnostic imaging, a practice guideline for spinal disorders state that conventional radiographs are not indicated for non-specific acute, sub-acute or persistent NP, in the absence of red flags [35]. Magnetic resonance imaging (MRI) is frequently advocated for the evaluation of NP as it can determine the presence of serious underlying disease, and is helpful in confirming the site and level of root compression. However, MRI of the cervical spine gives little attention to muscular tissue, apart from when utilised for research, which again may partly explain why it fails to identify these structural changes possibly related to symptoms [29]. Diagnostic ultrasound is safe, non- invasive, non-ionizing, with the ability to do measurements in real time. It is both cheaper and more cost-effective than MRI and easy to fit into a clinical setting in primary care dealing with musculoskeletal issues. However, it is known to be operator dependent [28, 36–38].

Javanshir et al. [70] reviewed 16 different studies of ultrasonography of the cervical muscles and argued that there was not good enough evidence to conclude that MSK-DUSI was appropriate for assessing neck muscles, even though previous studies had indicated it to be both reliable and valid [39–41]. For reliability studies, they suggested using constant landmarks, knowledge of anatomy and function of target muscles, and proper definition of muscular borders to help obtain a clearer image. Further, they highlighted the use of standardised subject positioning, the correct placement of the transducer, and the use of multiple images for statistical analysis in order to improve results. Thus there is a need for further investigation in order to determine the clinical utility of ultrasound in this area.

The main objective in this study was therefore to investigate one aspect of clinical utility by determining the inter-rater reliability and degree of agreement of determining the thickness of the Lcol, Rcpm, Dce and Sscap, by four raters on the same ultrasound image. It was also deemed important to ascertain the repeatability between days and between scans by investigating the intra-reliability of MSK-DUSI in measurement of the thickness of the same muscles.

## Methods

Intra- and inter-rater reliability and agreement study.

### Raters

Four raters, living in different parts of Norway, were involved in the study. The raters were chiropractors with at least 4 years of clinical experience in MSK-DUSI and had post-graduate certificates and diplomas in MSK-DUSI, from a CASE accredited University. This article is based on the four raters separate thesis submitted in partial fulfilment of the requirements leading to the degree of MSc Ultrasound. Their method was the same, but each rater had separate muscles to concentrate on. Beforehand the raters had agreed on which four muscles they considered most appropriate to investigate according to the literature describing muscle impairment in neck pain disorders [42], and these were then allocated by drawing lots to each of them. Hence each rater had one of the four muscles to scan and evaluate, but all four raters performed measurements on each other’s ultrasound images when they later met up in each other’s individual clinics.

### Study subjects

An a priori decision was made to include a total of at least 50 female subjects for each muscle investigated. This was considered an accessible size as the subjects were invited into the study from the four raters’ private chiropractic clinics from October 2014 to April 2015. The subjects were enrolled by consecutive invitation over the period of one to three months at each clinic. See details for inclusion/exclusion criteria in Table 1. All the participants gave verbal and informed written consent prior to study enrolment. Images and data were collected and stored anonymously. Application for ethics approval was first sent to the Regional Ethics Committee (REC) in Norway. They concluded that; “as this is not a collection of information related to health or illness, apart from NP, but rather a quality assurance of a diagnostic tool for cervical muscles, the project can be accomplish without approval from REC”.

**Table 1:** Inclusion and exclusion criteria

Inclusion criteria
• Adults (20–70 years of age) • Females • With or without neck pain
Exclusion criteria
• Diagnosis of recent trauma, spinal surgery, fractures or structural pathologies in the area of interest, to ensure the right anatomical landmarks on the ultrasound image during the ultrasound procedure of identifying the muscle • Not able to comply with the examination procedure • If for whatever reason anatomical landmarks and muscle borders could not be clearly enough identified on the ultrasound images for analysis

### Procedures

#### Ultrasound machine and scanning procedures

The ultrasound machines used were the one the raters had available in their clinics. For the Rcpm and Sscap, the Esaote MyLab5 ultrasound machine was used, while a Medison X 8 was used for Lcol and Dce muscles. The scanning was performed in B-mode. A linear probe applied with a 40 mm footprint and high frequency (12–18 MHz chosen individually) was used. The radiological principal ALARA (as low as reasonably achievable) was followed to obtain the necessary information, with minimal settings and examination time. No standard protocol (from EFSUMB or BMUS) existed for ultrasound scanning of the neck muscles. Thus, prior to testing, scanning procedures were based upon extended knowledge about anatomy from books and scientific articles. An excursion with a professor in anatomy at a dissection lab, including dissection and ultrasound scanning of a female cadaver, as well as training sessions and discussions within the study group were also a part of preparations prior to commencement of the study.

#### Thickness assessment of the Lcol muscle

**Fig. 1:**
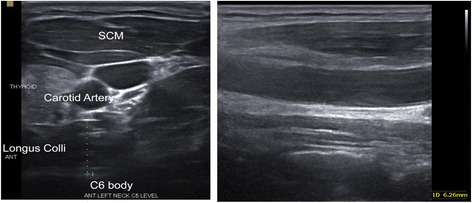
Ultrasound image of the longus colli at C6 in transverse view to the left behind the carotid artery, and to the right longitudinal where measurements were taken. The caliper is placed on the midpoint of the ventral surface of the C6 vertebral body and the interface between the Lcol and the pre-fascial tissue surrounding the carotid artery

Subjects were supine in a neutral position with a small towel placed under the neck to support the cervical lordosis, knees and hips were bent, and arms lying along the sides of the body. The thyroid and cricoid cartilages were palpated and the ultrasound probe was placed in the sagittal plane in the midline of these cartilages. The cricoid cartilage corresponded to the C6 level [43], while the bottom of the laryngeal prominence of the thyroid cartilage corresponded with the C5 level [40]. With the cricoid cartilage in the middle of the screen, the probe was moved laterally over the thyroid gland until the carotid artery was visible in the longitudinal view. The Lcol was then visible between the carotid artery and the vertebral bodies (VB). The thickness measurement of the Lcol was taken from the midpoint of ventral surface of the C6 vertebral body, defined as the posterior border of the muscle, to the ventral part of the muscle, at its border to the pre-facial tissues, surrounding the carotid artery (Fig. 1).

#### Thickness assessment of the Rcpm, Dce, and Sscap muscles

**Fig. 2:**
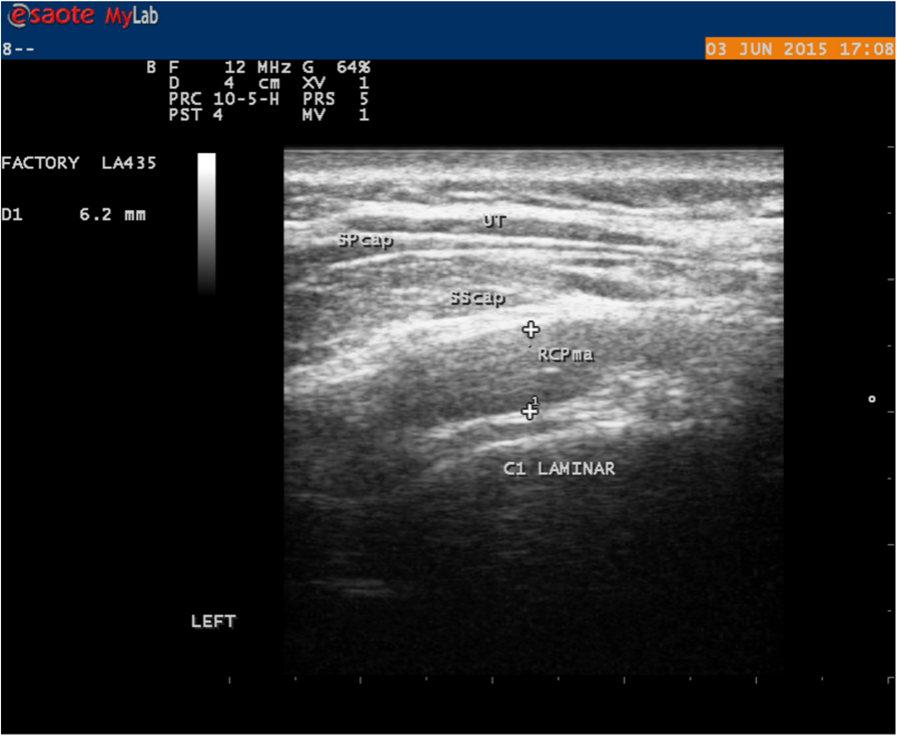
Ultrasound image of the Rcpm at rest in transverse view at C1. The caliper is placed around the midpoint above the C1 lamina betwen the inner and outer borders of the muscle where it was considered thickest by the raters

Subjects were placed in a prone position, as it has previously been shown to give more reliable measurements [61]. Foreheads were placed onto the adjustable headpiece of the bench with slight flexion of the head. The examiner was positioned on the left side of the subject. The Rcpm muscle is situated between the posterior tubercle of atlas to the medial region of the inferior nuchal line. It lies deep to the Sscap, splenius capitis and upper trapezius muscle. The C2 spinous process (SP) was identified by palpation. The transducer was placed transversely and moved laterally to identify the lamina at the C2 level. From this position the transducer was moved superiorly to identifying the lamina at the C1 level, where the examiner tilted the probe upward or downward to clearly identify the borders of the Rcpm muscle, in the transverse plane. The anterior-posterior dimension (APD) was used for thickness measurement of the muscle, taken at the largest distance between the inner and outer borders of the Rcpm muscle (Fig. 2).

**Fig. 3:**
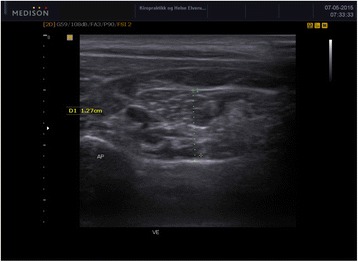
Ultrasound image of the Dce in transverse view at C5. The caliper is placed 90° to the lamina of C5 where the rater considered the muscle to be at its thickest and up to the echogenic line of the hyperechoic fascia between the Sscerv and Sscap

For the Dce, containing the semispinalis cervicis (Sscerv), the cervical multifidus (Cxmult), and the rotators, the transverse process on one side of C7 was identified in the transverse plane. The probe was moved medially to visualise the articular pillar and was then turned longitudinally, to identify the cervical facet joint of C5-6. The articular facet between C5-6 was placed in the middle of the image and the probe was again turned transversely. In this position, the lateral insertion of Cxmult on the articular facet and capsule was identified, while the probe was moved medially toward the base of the SP. The probe was angled up- or downwards to make the anterior and posterior borders as sharp as possible, before the image was captured. Thickness of the deep cervical extensors was determined as the measurement between the two echogenic lines of the lamina of C5 and the echogenic line of the hyperechoic fascia between Sscap and Sscerv. The calliper was positioned at 90° in relation to the laminae. The measurement was performed where the rater considered the muscular unit to be at its thickest (Fig. 3).

**Fig. 4:**
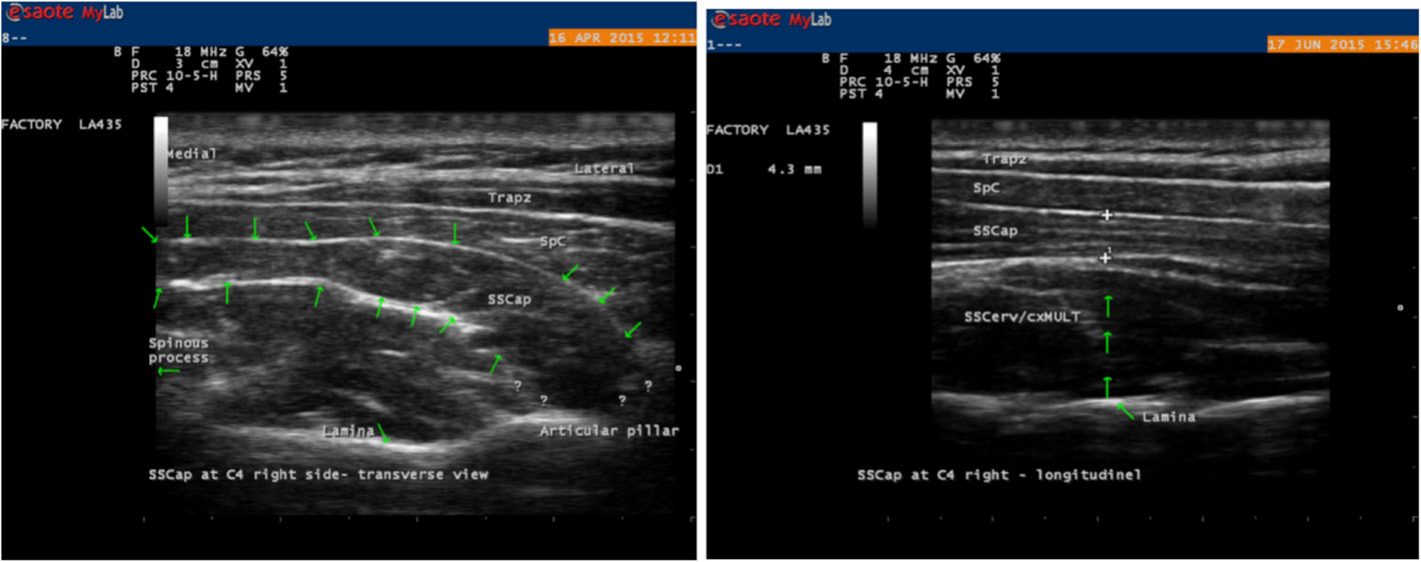
Ultrasound image of the Sscap at C4 in transverse view to the left for dynamic visualisation of anatomical borders and in longitudinal view where measurements were taken to the right. The caliper is placed from the lower border the Sscap against the Dce above the midpoint of the lamina of C4 to the outer border of Sscap against the Splenius Capitis muscle

Sscap, the third muscle in the layer, was recognised by its medial and lateral parts with aponeuroses visualised internally. To identify Sscap, the transducer was placed transversely to the level of C4. The bifurcation of the carotid artery usually occurs at the level of C4. The transducer was placed transversely to the SP and was moved laterally and anteriorly to identify the carotid artery on both sides, to ensure that the C4 level was in the image. The thickness of the muscle was measured by APD, at its thickest part over the midline of the lamina of C4, in the longitudinal view. An image in transverse plane was saved to decrease measurement error. This was achieved due to it giving dynamic visualisation of the fascia layers, thus clarifying which bright lines were actually within the muscle or fascial layer dividing the muscles (Fig. 4).

One image (image A) was obtained for each muscle from the left and right sides. Once the first two images were taken, the subject stood up from the bench, walked around and was re-positioned and the procedure was repeated for two more images (image B). In the intra-rater reliability part of the study, rater one measured the muscle’s thickness as image A was captured, and randomly again one week after all of the images had been collected. Thickness measurements performed by rater one on image B of the same subject, captured after re-positioning, were performed one week after all the data was collected. For the inter-rater reliability part of the study, the other raters measured muscle thickness on the entire A images and these were plotted together with the first measurements from rater one. Each of the raters’ measurements was compared pairwise to the other raters’ measurements. Thus in total, six ratings were collected for each muscle. This procedure was repeated for all of the muscles. The measurements were performed using the calliper software on the machine. The mean of two or more ratings has been recommended to increase reliability [49]. However, it was decided that only one single measurement for each image would be recorded, as this was considered more comparable to clinical practice. Still, when in doubt the raters were allowed a couple of measurements without recording them before determining what they considered to be the correct single measure. The measurements were recorded manually and not saved on the machine. The method was clarified to the raters prior to them undertaking the measurements. The raters were blinded for the subjects’ identification, clinical information and each other results. The results of the measurements were recorded manually on a list with the corresponding subject numbers, and transferred to an excel file for later statistical analysis. On the sheet of the ratings, comment fields were made available for the raters to comment on potential difficulties they encountered in performing the measurements.

### Data analysis

All of the data were analysed with IBM SPSS version 23 software. Descriptive statistics were used to describe the study population. The intra- and inter-rater reliability was analysed by calculation of the ICC, known as analysis of variance (ANOVA), which reflected both the degree of consistency and agreement among ratings. ICC was determined by a two-way mixed model, type absolute agreement, with a 95% CI (confidence interval), for single measures (ICC 3.1). For the estimation of the level of agreement and illustration of measurement error, the Bland-Altman plot was considered most relevant [44]. The raters were tested against each other using separate pairwise plots. A calculation of the pair-wise differences for the six comparisons was made and averaged together to define the y-axis. The x-axis was defined by the average mean of the measurements made by the four raters. Limits of agreement (LoA) (2 × SD) were calculated for each pair and a linear regression analysis were performed to evaluate for possible bias. Based on the LoA range we calculated the greatest difference % measured between examiners when applied to the average thickness of the different muscles.

## Results

### Descriptive data

A description of the study subjects is seen in Table 2. 50–56 different subjects were recruited for each muscle. Prior to analysis, 19 subjects for the Rcpm and 3 subjects for the Dce were excluded due to difficulties with landmarks and muscle borders and one for the Sscap as the splenius capitis muscle could not be identified.

**Table 2:** Descriptive data

Muscle	Females (N)	Mean age (Yrs)	Age range (Yrs)	SD (Yrs)	Neck Pain	No Neck Pain
Lcol	54	46,9	20–70	±13,4	61%	39%
Rcpm	31	44,1	20–70	±10,7	48%	52%
Dce	56^a^	50,0	20–70	±11,3	64%	36%
Sscap	50	37,1	20–70	±13,5	64%	36%

^a^6 of the subjects were excluded from to the inter- or intra-rater reliability and agreement analysis because of missing measurements due to difficulties defining anatomical borders

### Inter-rater reliability

Separate analyses were made for the right and left sides. As the results were similar, only the right side is reported. When measurements from the four raters were compared, the ICC values for the Dce and the Rcpm were moderate towards poor, and generally lower than for the Lcol and Sscap muscles, where inter-rater reliability was good, see Table 3. In addition confidence intervals for the Dce and Rcpm muscles were wider indicating greater uncertainty around the estimate.

**Table 3:** Inter-rater reliability - ICC values and corresponding confidence intervals

Muscle	Ex1-2ICC (95% CI)	Ex1-3 ICC (95% CI)	Ex1-4 ICC (95% CI)	Ex2–3 ICC (95% CI)	Ex2–4 ICC (95% CI)	Ex3–4 ICC (95% CI)
Right Lcol	.87 (.77–.93)	.90 (.83–.94)	.86 (.73–.92)	.84 (.72–.91)	.77 (.63–.86)	.84 (.70–91)
Right Rcpm	.86 (.71–93)	.54 (.23–.75)	.65 (.39–.81)	.46 (.14–.69)	.66 (.33–.83)	.43 (.11–.68)
Right Dce	.67 (.49–.79)	.54 (.32–.71)	.63 (.42–.77)	.62 (.43–.77)	.46 (.22–.64)	.54 (.32–.71)
Right Sscap	.88 (.72–.95)	.89 (.75–.95)	.87 (.77–.93)	.76 (.33–.89)	.85 (.75–.91)	.74 (.47–.86)

**Fig. 5:**
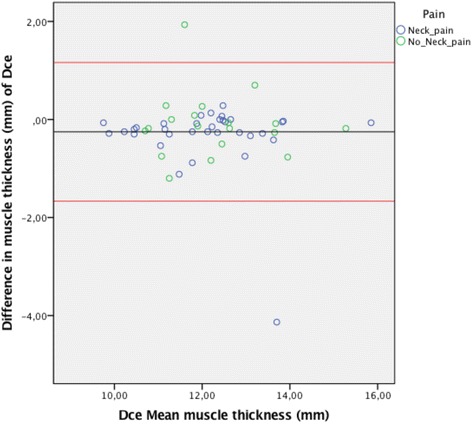
Bland & Altman Plot with LoA for the right Dce muscle showing agreement between the 4 raters for measurements of thickness on the same image. (*N* = 53 subjects, 212 ratings). The mean difference was calculated from the 6 pair of comparison of the different examiners and the mean thickness were calculated for the four raters

**Fig. 6:**
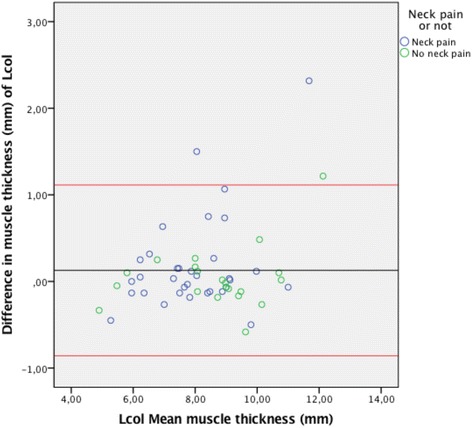
Bland & Altman Plot of LoA for the right Lcol muscle showing agreement between the 4 raters for measurements of thickness on the same image. (*N* = 54 subjects, 216 ratings). The mean difference was calculated from the 6 pair of comparison of the different examiners and the mean thickness were calculated for the four raters

**Fig. 7:**
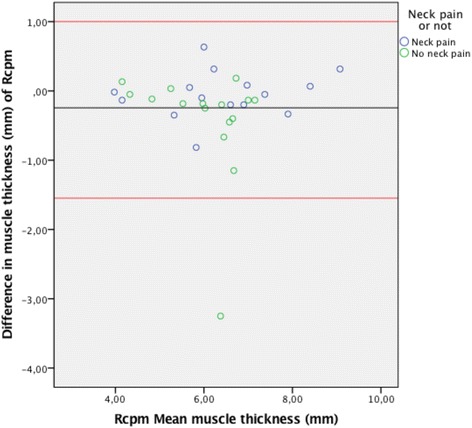
Bland & Altman Plot of LoA for the right Rcpm muscle showing agreement between the 4 raters for measurements of thickness on the same image. (*N* = 31 subjects, 124 ratings). The mean difference was calculated from the 6 pair of comparison of the different examiners and the mean thickness were calculated for the four raters

**Fig. 8:**
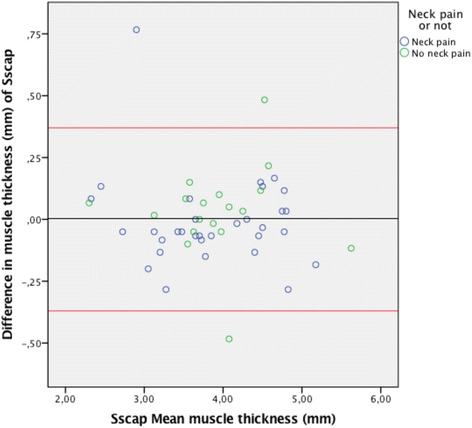
Bland & Altman Plot of LoA for the right Sscap muscle showing agreement between the 4 raters for measurements of thickness on the same image. (*N* = 50 subjects, 200 ratings). The mean difference was calculated from the 6 pair of comparison of the different examiners and the mean thickness were calculated for the four raters

The Bland-Altman plots for inter-rater agreement are shown in Figs. 5, 6, 7 and 8 and Table 4. The comparison of measured thickness between raters (Figs. 5, 6, 7 and 8) revealed a low mean difference, except for the Dce. For Dce the mean difference was approximately 20% of the average muscle thickness (2,52 of 12,2 mm), but only 2% (0.13 of 8,3 mm), 4% (0.24 of 6,2 mm) and 0.1% (0.003 of 3,9 mm) for Lcol, Rcpm and Sscap, respectively. The greatest difference % was considered as the maximal possible error of thickness measurement; which for the Dce was 14% (1,67 of 12,2 mm), 13% for Lcol (1,11 of 8,3 mm), 25% for Rcpm (1,55 of 6,2 mm) and 10% for Sscap (0,37 of 3,9 mm). Zero did lie within the LoA intervals, thus reflecting that there was no fixed bias. A linear regression analysis was performed for all the plots. The *P*-values were all > 0.05, so there was no proportional bias.

**Table 4:** Mean thickness, mean difference, agreement intervals (LoA) & linear regression analysis

Muscle	Mean muscle thickness (mm)	Mean difference (mm)	LoA range (mm)	Linear regression (p > 0.05)
Right Lcol	8.3 ± 1.6	0.13	(−0.86; 1.11)	0.06
Right Rcpm	6.2 ± 1.2	−0.24	(−1.55; 1.06)	0.91
Right Dce	12.2 ± 1.3	−2.52	(−1.67; 1.16)	0.46
Right Sscap	3.9 ± 0.7	0.003	(−0.37; 0.37)	0.58

### Intra-rater reliability

There was found to be good intra-rater reliability for all four muscles when measurements were done on the same image, see Table 5. However, when measuring on two different images of the same muscle the Dce showed poorer intra-rater reliability. The mean difference was found to be small, for both between days and between scan repeatability, but the agreement intervals were wider between scans. The greatest difference % measured by the examiner when applied to the average thickness of the different muscles was the same for Sscap and Rcpm, as in the inter-rater reliability part (10% (0,37 of 3,9 mm) and 25% (1,57 of 6,2 mm) respectively), and higher for Lcol (26% (2,14 of 8,3 mm)) and Dce (22% (2,66 of 12,2 mm)).

**Table 5:** Intra-rater reliability and agreement -ICC values and corresponding confidence intervals and mean difference with agreement intervals

Muscle	Repeatability between days - same image measured on two separate occasions		Repeatability between scans – measurement of two different images of the same muscle	
	ICC (95% CI)	Mean difference (95% LoA)(mm)	ICC (95% CI)	Mean difference (95% LoA)(mm)
Right Lcol	0.97 (.94–.98)	0.00 (−0.90; 0.90)	0.82 (.70–.89)	0.03 (−2.07; 2.14)
Right Rcpm	0.95 (.89–.97)	0.17 (−0.73; 1.95)	0.86 (.75–.93)	−0.17 (−1.57; 1.23)
Right Dce	0.81 (.70–.89)	0.02 (−1.70; 1.74)	0.52 (.29–.70)	−0.11 (−2.66; 2.44)
Right Sscap	0.97 (.95–.99)	−0.01 (−0.34; 0.32)	0.95 (.91–.97)	0.02 (−0.37; 0.37)

## Discussion

The aim of this study was to establish the reliability of MSK-DUSI in measuring the thickness of the Lcol, Rcpm, Dce, and Sscap by four clinicians. The lower reliability found for the Rcpm and Dce may be because morphological changes, as fat infiltration in the deepest extensor muscles including the Rcpm, may make anatomical landmarks and muscle borders more difficult to define. The ICC values were higher in the intra-reliability part of the study, which was expected as the same rater who took the images also did the measurements, and hence probably had a greater understanding of that muscle and its borders. For a test to be useful on a consistent basis in clinical practice reproducibility would be considered of more importance than repeatability [45]. However, if the inter-rater reliability is poor, knowledge of the intra-rater reliability might assist in identification of sources of error, as may be the case in this study.

Statisticians maintain that one should not seek agreement between different methods or measurers; instead one should focus on disagreement or bias [46]. This study therefore focused on this school of thought. However, a priori definition of acceptable limits for the agreement interval based upon clinical necessity and biological considerations, as proposed by Giavarina [71], had not been made. In general, the mean difference between raters was low, except for the Dce muscle. If we were to use ultrasound for diagnostic purpose, such as follow-up measurements, any reported change above the mean difference may be associated with actual change in a muscle and not be a result of the reliability of the measurement method. Still, the agreement interval indicated a measurement error range that appeared to be too high considering the size of theses muscles and probable changes seen in relation with NP. LoA has not often been reported in comparable previous studies, and to our knowledge, no previous literature has yet outlined acceptable agreement levels for these muscles.

### Methodological considerations - strengths and limitations

To improve the quality of the study, the methodology employed was tailored according to proposed recommendations in the literature [48–50]. It has been highly recommended for reliability studies that they reflect the circumstances in which they would like the results to be generalised [50]. This study included a representative sample from a typical clinical setting in primary care. The sample size was generally higher than that used in previous studies. The current study included a wider age range and was thought to be large enough to represent a variety of different subject types, as well as subjects with and without neck pain. Only females were included, which made the population more homogenous, an important criterion for reliability studies.

Unlike most previous studies both the ICC and the B&A test with LoA were used. An advantage of the B&A plot is that the graph provides a representation of the magnitude of the degree of agreement. One can easily identify bias, outliers, and other relationships between the variance in measure [51].

The small thickness of these muscles may have amplified errors, thus influencing the variability of measurements [33]. With lack of variability, measurements might have fallen within a restricted range that could also have affected the ICC [52]. Four raters were available for the inter-rater part of this study, thus allowing one to yield a more precise reliability estimate. This were considered a strength of this study, even though the numbers of subjects used was thought to have a greater impact on the accuracy of the results than the number of raters [48]. Owing to the use of more than two repeated measurements, calculations were more complex. As a result, the sample size needed to be large enough, preferably greater than 50, to allow the B&A’s LoA to be estimated and to avoid the CI becoming too wide [51]. Even though this study included more subjects than most of the previous studies in this topic area, the sample may still not have been sufficiently large enough when 4 raters were included [47]. Reliable results of small muscles have also reported to be challenging using MRI, despite it being regarded as the gold standard [40]. However, no validated method existed to quantify atrophic changes and fatty infiltration with MSK-DUSI, as the Goutallier classification system on MRI [53].

As the cervical muscles are complex and anatomy may vary between individuals, differences in consistent anatomical landmarks represented a challenge. Measurements were only taken at one spinal level, considered consistent for each muscle. The images were two dimensional (2D), so the entire muscle could not be visualised. It was also challenging to reproduce the muscle image in the exact same plane. There were issues regarding accurate documentation of tissue boundaries and anatomical landmarks. Either because the transition between the different muscles layers was blurred or because of thickened fascia and aponeuroses were difficult to distinguish from each other. A cause for this might be muscular degeneration where decreased water content and increased fat and fibrous content may give a greater echogenicity and change in the architectural features of muscles [28, 54]. These changes could have affected the interpretation of the images in this study, as several images had to be excluded, especially for the deep Dce and the Rcpm. Rankin [55, 56] have also reported the same difficulties in image interpretation. Degenerative changes as osteophytes could have developed in the cervical spine of the subjects and made the bony landmarks more difficult to define on the ultrasound images. Along the superior border of Lcol, is a fascial layer containing the superior ganglia and in some patients this layer also contained a blood vessel. Similarly in the transverse view of the Sscap, a vein was sometimes seen lying between the Sscap and the Sscerv/Cxmult (most likely the deep cervical vein - a branch from the vertebral vein). This was an important consideration, as these vessels could have easily been mistaken as being part of the muscle in the longitudinal view, particularly as Doppler was not standardly used. To help counteract this, the transverse image was taken to help define these borders for the Sscap only. Despite this consideration, several of the images were reported to have uncertain muscle borders. None of these were removed from the analysis. For all the muscles in this study it was decided to measure the APD, as measuring muscle thickness tend to yield lower levels of measurement error compared to CSA [50]. For the Lcol, it may be challenging to define its medial border, due to the shadowing of the trachea, when measuring CSA or its lateral dimensions [40]. On the other hand, the muscle might not have been captured at its thickest part or the exact same location, as the APD was measured on longitudinal images. Transverse images may visualise this better, but it is thought to be more difficult to confirm the exact levels where the measurements are taken on transverse images. The Rcpm was captured in transverse plane, in order to allow comparison with a previous study by Lin et al. (2009) [57]. Longitudinal images might have improved the identification of muscle borders for this muscle. The Dce was measured using the APD and as a group. Although this differed from the methodology utilised in previous studies [39, 58, 59], this decision was based on recommendations made by these studies. It was found to be near impossible to distinguish this group of muscles individually, both on a pre-study cadaver investigation and on MSK-DUSI. However, the Dce was captured transversely. Using a longitudinal view would not have captured this muscle completely due to its oblique course and varying angulation of the muscle fascicles [21]. The Sscap has been described as a complicated muscle, due to tendinous inscriptions and internal aponeuroses that interrupt fascicles and can ultimately lead to underestimation of the CSA of the muscle [60]. Its boomerang shape made it difficult to outline the borders of the whole muscle, especially lateral to its aponeurosis. According to Stokes et al. (2007), longitudinal images may be easier to interpret than transverse views, both for measuring muscle thickness and for providing biofeedback of potential changes in the muscle during contraction [33]. Ideally in conclusion, an orthogonal view (both longitudinal and transverse views) for all muscles should be used in order to enable optimal visualisation.

### Comparison with previous studies

It is difficult to directly compare previous studies with the current study, primarily due to methodological differences. In these studies, the ICC has often been considered, more often intra-rater reliability of measuring muscle size of various cervical muscles, with a range from 0.60–0.99 [39, 40, 55, 59, 61–64, 70]. In general, fewer subjects have previously been included, with the recruited subjects often being younger and healthy with no NP [47, 55, 57, 59, 62, 64]. The spinal level investigated has differed in previous studies [15, 40, 55, 58, 59, 61–63, 65–67]. Few previous studies have looked at agreement. There have also been issues with blinding not being accounted for [55, 57, 61], and incomplete reporting on reliability [39, 51, 58], which limits the generalisability of their findings.

### Recommendations for future studies

We are uncertain whether further improvement of measurement procedures or more training will allow the raters to better agree and reduce errors, especially on images featuring difficult anatomical borders unless new technology with use of different ultrasound apparatus applications can improve this. Nevertheless, our study has provided clinicians with a recommendation for an ultrasound scanning protocol and measurement procedure for four different neck muscles. Intra-rater reliability was greater than inter-rater reliability and therefore our recommendation so far would be that the same examiner performs all ultrasound examinations, especially if repeated exams are being performed on each individual. We recommend the use of orthogonal views. Access to a video clip of the scanning may also be useful. More anatomical landmarks than the SPs should be used to identify cervical levels, as described in the methods, as differentiation otherwise may be difficult. However, the validity of this must be investigated further. The levels of investigation may also need to be reconsidered depending on muscle function at different spinal levels. In a study by Skeie et al. (2015), grading the degree of contraction of the lumbar multifidus, not measuring the exact thickness change, was suggested [68]. This may be a more clinically relevant approach that may improve the agreement intervals in future studies. The Sscap and the Dce may be considered a functional unit as they span lateral to medial as the transverso-spinal system and all act as agonists with common neural signals [19, 72]. Hence they could be evaluated with MSK-DUSI as a group, particularly as they are small muscles when considered individually. This may decrease the measurement error, relative to the muscle thickness, and tissue borders may be easier to interpret. If the whole unit is evaluated, the degree of contraction can be categorised. This of course presumes that all layers respond equally in various neck disorders, which may not be the case. Future studies should aim to establish what constitute clinically relevant muscle changes, and to outline acceptable agreement levels for these muscles. As it is recommended that future studies assess other functionally muscular related variables that pertain to muscle morphology [69], it would be interesting to see if MSK-DUSI could be a reliable method in quantifying the degree of muscle atrophy and fat infiltration of cervical muscles, and whether this has any clinical value. Implementation of ultrasound into clinical practice, may in the future, act as an objective tool in the evaluation of neck pain, but can not at present be considered appropriate for clinical use.

## Conclusion

The results of this study suggest that MSK-DUSI, as an imaging tool to assess the thickness of various neck muscles, had good inter-rater reliability for Lcol and Sscap muscles and moderate towards poor for the deeper Rcpm and Dce muscles when tested by experienced raters on females with and without NP. Intra-rater reliability was found to be good for all the muscles, except for the Dce, which was moderate towards poor, for between scans repeatability. However, the agreement intervals indicated measurement errors within this method for all muscles that probably are not acceptable, especially if one should look for thickness changes in clinical practice or clinical studies. Future enhancement of ultrasound technology may solve some of the challenges with defining anatomical landmarks and tissue variables, and hence improve both the ICC and the LoA.

## Abbreviations

APD: Anterior-posterior dimension
B&A: Bland & Altman
BLM: Birgitte Lawaetz Myhrvold
CI: Confidence interval
CKØ: Cecilie Krage Øverås
CSA: Cross-sectional area
Cxmult: Cervical multifidus
Dce: Deep cervical extensors
EM: Eli Magnesen
GR: Gro Røsok
ICC: Intra-class correlation coefficient
Lcol: Longus colli muscle
LoA: Limits of agreement
MRI: Magnetic resonance imaging
MSK-DUSI: Musculoskeletal diagnostic ultrasound imaging
NP: Neck pain
Rcpm: Rectus capitis posterior major muscle
REC: Regional ethics committee
Sscap: Semispinalis capitis muscle
Sscerv: Semispinalis cervicis muscle, Sp, spinous prosess
Tp: Tranverse prosess
